# 3D printing of cellulose nanocrystals based composites to build robust biomimetic scaffolds for bone tissue engineering

**DOI:** 10.1038/s41598-022-25652-x

**Published:** 2022-12-08

**Authors:** Kanga Marius N’Gatta, Habib Belaid, Joelle El Hayek, Edja Florentin Assanvo, Marilyn Kajdan, Nathalie Masquelez, David Boa, Vincent Cavaillès, Mikhael Bechelany, Chrystelle Salameh

**Affiliations:** 1 Institut Européen des Membranes, IEM, UMR 5635, Univ Montpellier, ENSCM, CNRS, Montpellier, France; 2 Laboratoire de Thermodynamique et de Physico-Chimie du Milieu, UFR SFA, Université Nangui Abrogoua, 02 BP 801, Abidjan 02, Côte d’Ivoire; 3 IRCM, Institut de Recherche en Cancérologie de Montpellier, INSERM U1194, Université Montpellier, 34298 Montpellier, France

**Keywords:** Biomedical engineering, Bioinspired materials, Biomineralization, Tissues, Biomaterials

## Abstract

Cellulose nanocrystals (CNC) are drawing increasing attention in the fields of biomedicine and healthcare owing to their durability, biocompatibility, biodegradability and excellent mechanical properties. Herein, we fabricated using fused deposition modelling technology 3D composite scaffolds from polylactic acid (PLA) and CNC extracted from *Ficus thonningii*. Scanning electron microscopy revealed that the printed scaffolds exhibit interconnected pores with an estimated average pore size of approximately 400 µm. Incorporating 3% (w/w) of CNC into the composite improved PLA mechanical properties (Young's modulus increased by ~ 30%) and wettability (water contact angle decreased by ~ 17%). The mineralization process of printed scaffolds using simulated body fluid was validated and nucleation of hydroxyapatite confirmed. Additionally, cytocompatibility tests revealed that PLA and CNC-based PLA scaffolds are non-toxic and compatible with bone cells. Our design, based on rapid 3D printing of PLA/CNC composites, combines the ability to control the architecture and provide improved mechanical and biological properties of the scaffolds, which opens perspectives for applications in bone tissue engineering and in regenerative medicine.

## Introduction

The development of materials for bone engineering remains a challenge due to the complexity of the natural bone structure and the biomechanical environment. To repair damaged bone tissue, autografts from different bones are harvested and used to replace missing bones. Less available allografts are discouraging^1–3^, and artificial junctions used as implants often have to be removed after healing^4^. Recently, new bone repair strategies have emerged, including scaffold-assisted regenerative medicine used to promote bone growth^5^.

An ideal bone scaffold should be a three-dimensional matrix capable of mimicking the complex composition and structure of the bone for cell attachment and proliferation^6^. Therefore, it requires high biocompatibility, biodegradability, non-toxicity, excellent mechanical properties and appropriate architecture in terms of porosity and pore sizes to integrate with native host tissue^7^. The chemical composition and physicochemical characteristics of the scaffold, directly influencing the mechanical and biological performance^8^, are therefore important parameters to study.

Synthetic biopolymers have been widely used in bone tissue engineering due to their biocompatibility and their ability to control the physicochemical properties of the scaffold. They consist of aliphatic polyesters such as polyglycolic acid (PGA), polycaprolactone (PCL) and polylactic acid (PLA)^9^. Unfortunately, they are rather brittle and usually lose their strength due to rapid degradation in vivo. Moreover, their hydrophobic nature hinders the attachment and proliferation of bone cells^10^. To overcome these limitations, scaffolds based on synthetic polymers, namely PLA or PCL can be improved by incorporating natural polymer reinforcements such as cellulose^11–15^, alginate^16^, gelatine^17^, chitosan^18,19^ or keratin^20^ known for their interesting characteristics.

Several techniques, including solvent casting and particle leaching, emulsion freeze-drying, phase separation, or electrospinning^21–23^, are used to develop scaffolds for hard tissue engineering. However, they do not allow efficient control of the morphology and porosity.

Additive manufacturing has proven to be a technique of choice for designing and preparing biomimetic bone repair materials. 3D-controlled scaffold architectures significantly affect mechanical properties as well as bone cell adhesion and proliferation^2,24–28^. Therefore, various works have focused on the development of 3D printed scaffolds using various technologies, such as stereolithogaphy, 3D plotting, selective laser sintering, bioprinting, and fused deposition modelling (FDM). FDM is the most widely used additive manufacturing technology. It is a simple and fast technique at low cost offering great possibilities for handling polymers.

Several studies have investigated the reinforcement of PLA with nanomaterials such as graphene oxide^29^, boron nitride (BN) nanofillers^21^, nano-hydroxyapatite^30–32^ to improve the mechanical and bioactive properties of the polymeric scaffold. Cellulose is a promising material for biomedical applications, including tissue engineering, stem cell research and regenerative medicine^33–37^ due to its excellent biocompatibility, favorable biodegradability, good mechanical properties, high specific surface area, low density and non-abrasive nature^38–41^. In particular, cellulose nanocrystals (CNC) are effective to be applied as reinforcing agents or nanofillers in scaffold design and the results confirmed that CNC significantly improve the mechanical performance and cytocompatibility of scaffolds^38,39,42,43^. In addition, CNC have many hydroxyl groups which can form intermolecular hydrogen bonds with carbonyl groups of PLA to achieve favorable interfacial adhesion. Blending PLA with biopolymers such as cellulose improves the properties of PLA without altering its biocompatibility. PLA/cellulose composites have been widely developed and studies on structural, thermal and mechanical properties have been reported^12,44–51^. However, even though PLA and cellulose nanocomposites are reported in the literature for tissue engineering applications, we believe that there is still room to develop 3D scaffolds for tissue engineering by FDM which is a rapid and low-cost additive manufacturing technique especially to overcome certain limitations encountered in the PLA, namely the mechanical resistance of the biopolymer.

This work therefore aims to unravel the effect of CNC content on PLA/CNC scaffolds processed by FDM and its influence on the final structural, surface and mechanical properties of the biocomposites. Biomineralization, in vitro biodegradability and cytocompatibility tests were performed on the resulting PLA/CNC3 (i.e. with 3% (w/w) CNC incorporated into the composite). The resulting material is a mechanically enhanced scaffold that offers great opportunities for the design and rapid production of biomimetic 3D scaffolds with appropriate biological properties.

## Results and discussion

### Characterization of PLA and PLA/CNCx filaments

**Figure 1:**
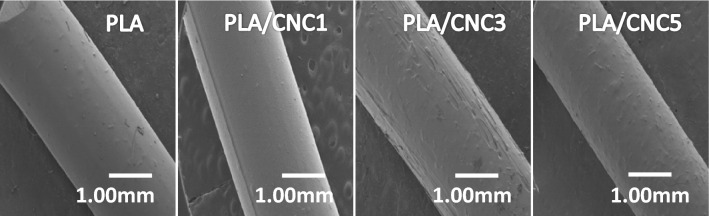
SEM images of extruded PLA and PLA/CNCx composite filaments.

Scanning Electron Microscopy (SEM) images reported in Fig. 1 clearly show pure PLA filaments with a smooth and regular surface while those of the composites are rough due to the formation of aggregates related to the presence of CNC. Indeed the evolution of the PLA surface, after the incorporation of CNC, from a smooth state to a rough state could suggest a good dispersion of CNC over the entire surface of PLA. The incorporation of more than 3% (w/w) of CNC results in the formation of large particles, which could lead to less hydrogen bonds between the matrix (PLA) and the reinforcement (CNC). Indeed, the dispersion of CNC in PLA is low due to the hydrophilic nature of cellulose *versus* the hydrophobic nature of PLA rendering their interaction and uniform dispersion difficult^52^.

The composite filaments, resulting from the hybridization of PLA, exhibit good capacities for improving cell binding and proliferation. In this respect, PLA/CNC composites are good candidates for biomedical applications, particularly in bone regeneration. Indeed, cellulose fillers (CNC) could create important cell attachment and proliferation sites for any implantable scaffold material in hard tissue engineering. Several studies have already been carried out to develop PLA/CNC composite filaments by single-screw extrusion printable by FDM^52–54^. However, the development of PLA/CNC filaments for the production of hybrid scaffolds for biomedical applications remains new to our knowledge.

Fourier-transform infrared spectroscopy (FTIR) analysis of the filaments are shown in Figure S1 in Supplementary Information. Our results show a slight increase in peak intensities with the increase of the CNC loading within the PLA matrix, which is in agreement with the literature^52^. The most significant differences were observed at 2946, 1743, and between 1500 and 1000 cm^−1^ which are respectively attributed to stretching of CH, C=O bonds, carbonyl stretching vibration (C=O) and to CO of PLA^55^.

**Figure 2:**
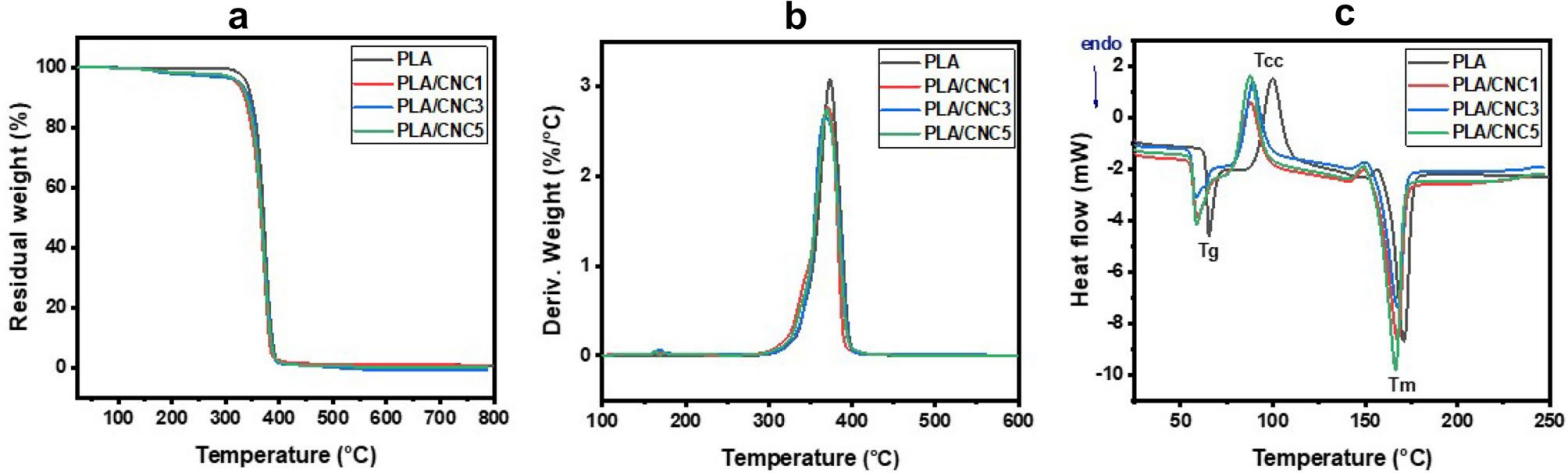
Thermal analysis of PLA and PLA/CNCx extruded filaments. (**a**) thermogravimetric analysis (TGA, under nitrogen from room temperature to 800 °C at a heating rate of 10 °C min^−1^), (**b**) derivative TGA (DTG) and (**c**) differential scanning calorimetry (DSC curves, from room temperature to 250 °C with a heating rate of 20 °C min^−1^ under nitrogen atmosphere) showing the T_g_, T_cc_ and T_m_ of PLA and the nanocomposites.

The decomposition of CNC, PLA filaments and the derived composites was examined from the thermogravimetric analysis (TGA), derivative thermogravimetry (DTG) and differential scanning calorimetry (DSC) curves reported in Fig. 2. A slight weight loss is observed between 100 and approximatively 325 °C in all samples due to the dehydration of filaments and depolymerization of PLA as well as the breaking of glycosidic bonds in cellulose. Compared to PLA, the composites exhibit higher weight loss due to the hygroscopic nature of the composites after incorporation of hydrophilic CNC. Therefore, when subjected to thermal stress, composites release the absorbed moisture, resulting in this relatively high weight loss. In addition, a strong weight loss occurs at 400 °C probably related to the degradation of PLA and CNC. The onset decomposition temperatures of CNC and the filaments are observed at 225, 318.8, 311.8, 325.5 and 320.4 °C for CNC, PLA, PLA/CNC1, PLA/CNC3 and PLA/CNC5, respectively. The developed filaments can therefore be processed at temperatures reaching 300 °C without damaging them. Therefore, the printing temperature of 200 °C used in our study, seems to be optimal for developing PLA/CNC scaffolds. However, CNC do not significantly improve the thermal stability of PLA. Moreover, the DTG curves show a drop in the maximum degradation temperature of the composites compared to that of pure PLA. The maximum degradation of PLA/CNC3 occurs over a temperature (T) range of approximately 365–374 °C while that of PLA, PLA/CNC1 and PLA/CNC5 occurs at T = 373.39, 370.62 and 369.22 °C, respectively. Indeed, the relatively wide degradation temperature of PLA/CNC3 can be attributed to the better hybridization of the composite reflected by a better interaction between 3% of CNC and PLA.

Furthermore, the analysis of the DSC curves (Fig. 2c) of all the samples successively reveals endothermic stage changes (glass transition), followed by an exothermic peak dedicated to cold crystallization and an endothermic peak due to the fusion. Compared to PLA, the composite filaments show a shift of the glass transition, cold crystallization and melting peaks towards the lowest temperatures. This suggests that the CNC tends to decrease the change-of-state temperatures of PLA (Table 1). Indeed, the glass transition range widens with the increase of CNC loading. This suggests that the addition of cellulose increases crystallinity due to the enhanced mobility of the PLA chains^56^. This is consistent with the calculations of the degree of crystallinity presented in Table 1. The addition of 3% (w/w) CNC into the PLA matrix caused a wider glass transition range with a weaker peak, which indicates better interaction between CNC and PLA and therefore could reflect a good dispersion and good interfacial adhesion.

**Table 1:** Glass transition temperature (Tg), cold crystallization temperature (Tcc), melt temperature (Tm), cold crystallization enthalpy (*∆H*cc)) and degree of crystallinity (${\chi }_{c}$) for PLA and PLA/CNCx composites from DSC.

Sample	T_g_ (°C)	T_cc_ (°C)	T_m_ (°C)	*∆H*_cc_ (J/g)	${\chi }_{c}$(%)
PLA	66	100	171	24	26
PLA/CNC1	57	88	168	20	22
PLA/CNC3	56	89	167	22	27
PLA/CNC5	57	87	166	23	26

In the Supplementary Information, Figures S2 and S3 display the thermal analysis of CNC and of 3D printed PLA and PLA/CNC3 respectively, which proves that PLA exhibits no thermodegradability when printed.

### Mechanical properties of 3D printed scaffolds

Implantable scaffolds for biomedical applications require specific attributes to best perform native organ functions and help regenerate damaged tissue. Among their attributes, the mechanical properties remain the most crucial to support the regeneration process. Several authors have investigated scaffolds combining various materials to provide successful substitution or mechanical support needed to promote new tissue growth at defect sites. However, the mechanical properties of hybrid scaffolds depend on the method of manufacture, the content, the orientation of the nanomaterials, the nature of the matrix, the interactions between the polymer matrix and the nanomaterials, etc^21,29,57–60^.

**Figure 3:**
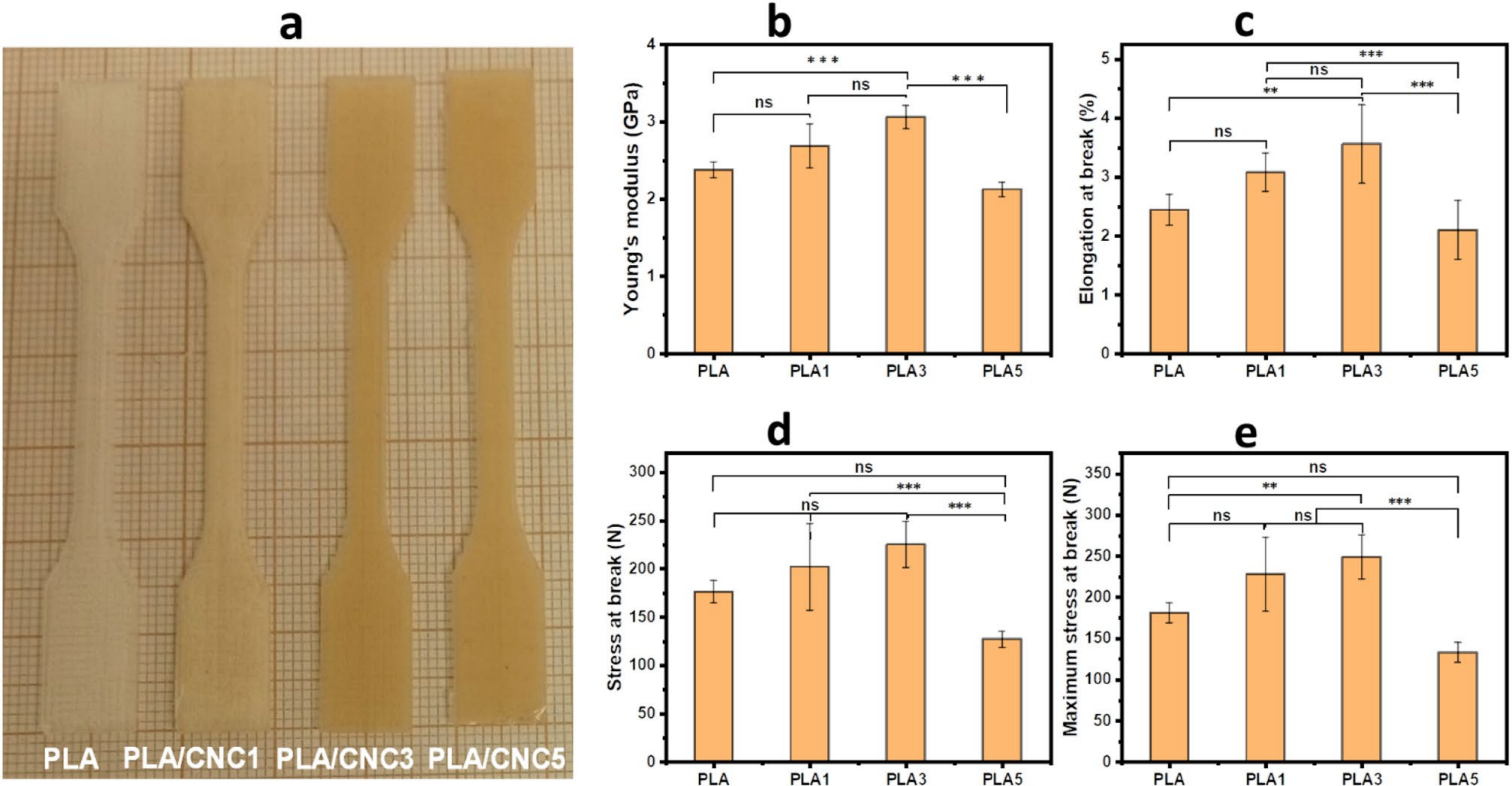
Mechanical properties: (**a**) Photo of dog bone shaped PLA and PLA/CNCx scaffolds for tensile strength testing. (**b**) Young’s modulus. (**c**) Elasticity limit tensile. (d&e) stress at break (***p ˂ 0.001, ** *p* < 0.01, ns = not significant).

Different filament compositions were used for 3D printing of PLA/CNC scaffolds using FDM as described in the experimental section. The analysis of the effect of cellulose on Young's modulus, elastic limit and strain at break of the 3D printed scaffolds was studied and the results are shown in Fig. 3. PLA scaffolds have an average Young's modulus of 2.4 ± 0.1 GPa in accordance with the literature^29,61^ and an average elongation at break of 2.4 ± 0.3% while the average Young's modulus and elongation at break of the composites range between 2–3 GPa and 2–3.6%, respectively. This is in good agreement with the properties of PLA/natural fiber composites^62^. It seems that the addition of 3% of CNC enhances the mechanical strength of PLA (*p* ˂ 0.001). This improvement may be due to better hybridization of CNC particles in PLA chains to ensure the best compatibility and absorb any external load. Indeed, at a relatively low concentration of cellulosic reinforcement, the molecular interactions between PLA and CNC provide good rigidity to the composites. This results in an improvement in Young's modulus^63^ from 2.4 ± 0.1 GPa for PLA to 3.1 ± 0.2 GPa for PLA/CNC3 because at low concentrations of CNC the particles tend to align in the direction of the orientation of the PLA chains^52^. Consequently, the applied stress is transferred from one fibril to the next within the composite structure, thus allowing a uniform distribution of the stress in the material. The improvement in elongation at break and deformation forces at break of the composites with cellulosic reinforcement ≤ 3% suggests that the presence of CNC tends to improve the elastoplasticity of PLA. This could result from the good fiber-matrix interaction due to a better interlocking of the CNC network in the PLA matrix. The incorporation of 5% of CNC tends to form large particles distributed in the composite, which results in the formation of fewer bonds between the matrix (PLA) and the reinforcement (CNC) and poor compatibility resulting in poor mechanical performance.

Based on these results, we focused the following analyses on PLA/CNC3 only. Indeed, when the CNC content is more than 3% (w/w), the PLA cannot sufficiently wet the surface of the CNC, resulting in strong fiber–fiber interaction and/or fiber agglomeration in the PLA matrix^52,54^. However, *Kumar *et al*.*^52^ found a 50% increase in the elastic modulus (E) with 1% CNC (4550 MPa) compared to pure PLA (3030 MPa). The elastic modulus of PLA and its composites was better than ours. Nevertheless, there is a decrease in the strain at break observed for 1% CNC (2.8%) compared to the pure PLA sample (8.7%).

### Morphology of 3D printed scaffolds

**Figure 4:**
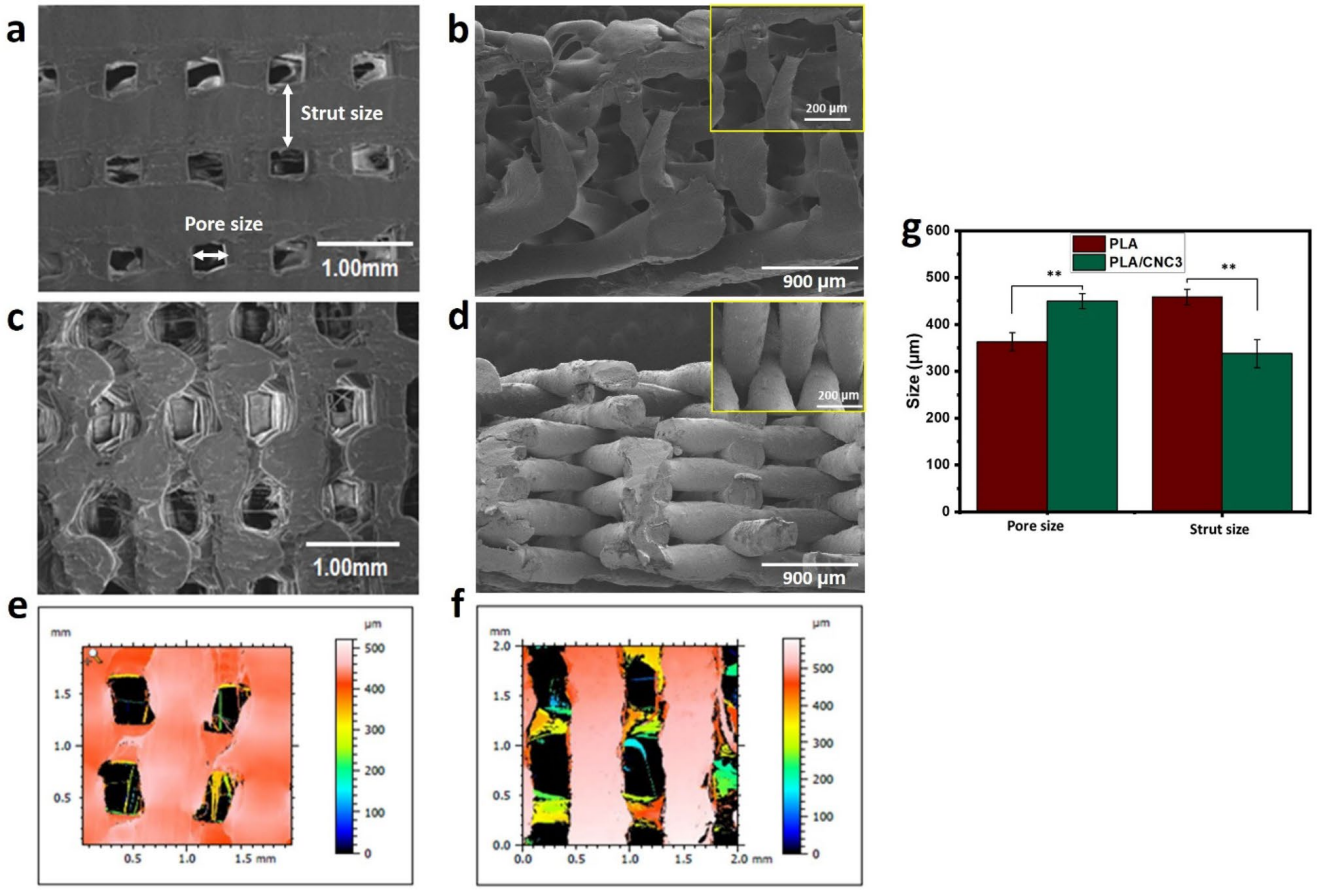
Scanning electron micrographs of 3D printed scaffolds: Top view of the morphology of (**a**) PLA and (**c**) PLA/CNC3. Cross-section with a zoom in inset of (**b**) PLA and (**d**) PLA/CNC3. (**g**) Statistical analysis of the 3D printed PLA and PLA/CNC diameters observed on top of the samples by SEM (Test Anova, **p < 0.01). Topography of 3D printed scaffolds typically showing the surface roughness of (**e**) PLA and (**f**) PLA/CNC3.

The surface morphology of PLA and PLA/CNC3 scaffolds was analyzed by SEM, 3D optical microscopy and topography (Fig. 4). These images clearly show that the PLA scaffold has a smooth surface while that of PLA/CNC3 is rough due to the presence of cellulose. Ideally, the printed scaffolds should have pores of the same size. However, analysis of SEM images (Fig. 4) reveals that the pore size of the hybrid scaffold (PLA/CNC3) increased while the width of the rods decreased similarly to what is reported in the literature^21,29^. In our work, the average pore size and the rod width of PLA and PLA/CNC3 scaffolds are respectively evaluated at (362 ± 19 µm and 458 ± 17 µm) and (450 ± 15 µm and 337 ± 30 µm). This variation in the morphology of the scaffold surface can be attributed to the modification of the rheological behavior of the matrix by the addition of CNC. Indeed, the addition of CNC could limit the elastic behavior of PLA and therefore give it stability to retain its shape during deposition. However, the reinforcement produces an instability of the material flow during printing probably affecting the quality and resolution.

Moreover, porosity is one of the most critical parameters to evaluate the efficiency and applicability of biomedical scaffolds, particularly in bone tissue engineering, drug delivery, etc. A high porosity promotes the efficient release of biofactors including cells, genes and proteins and provides an environment conducive to nutrient exchange^64,65^. The porosity of PLA and PLA/CNC3 scaffolds is relatively high (58 ± 2% and 67 ± 2%), respectively. The addition of 3% CNC increases the porosity of PLA scaffolds (**p ˂ 0.01), which can be attributed to the ionic interaction between the different components creating more voids in the scaffolds. With such porosity profiles, printed scaffolds could help improve bone ingrowth by allowing optimal vascularization, hydroxyapatite nucleation and mineral maturation^66,67^.

### Contact angle and swelling of 3D printed scaffolds

Hydrophilicity is a property that significantly affects the adhesion, cell proliferation and rehydration capacity of scaffolds; therefore, it appears crucial for the performance of scaffolds for any application in tissue engineering including bone regeneration. Thus, the reactivity and interaction between the printed scaffolds and the surrounding surface could be predicted by measuring the contact angle and studying the swelling in water. Figure S4 (see Supplementary Information) illustrates the water contact angles of PLA and PLA/CNC3 scaffolds and the values are presented in Table 2. CNC brings a hydrophilic character to the hydrophobic surface of PLA scaffolds due to the rearrangement of CNC in the PLA matrix. To better understand the effect of cellulose filler on the hydrophilicity of PLA, we evaluated the water absoprtion rate of scaffolds (Table 2). The addition of CNC leads to an improvement in the water uptake of PLA according to the results of the contact angle and surface roughness of the scaffolds. Similar water retention results were observed by *Murphy *et al*.*^56^ on PLA and PLA/cellulose filaments. They revealed a significant increase in water uptake during the first 24 h followed by a stabilization to gradually reach a plateau (1.2% for the PLA filament and 1.4% for the PLA/cellulose composite filament).

**Table 2:** Contact angle and swelling index of PLA and PLA/CNC scaffolds.

Materials	PLA	PLA/CNC3
Contact angle (°)	101 ± 1	84 ± 2
Swelling index (%)	4.1 ± 0.3	9.8 ± 0.7

In view of these results, PLA/CNC scaffolds are capable of creating an environment favorable to cell proliferation and therefore appear as the composites of choice for bone regeneration.

### Biodegradation

**Figure 5:**
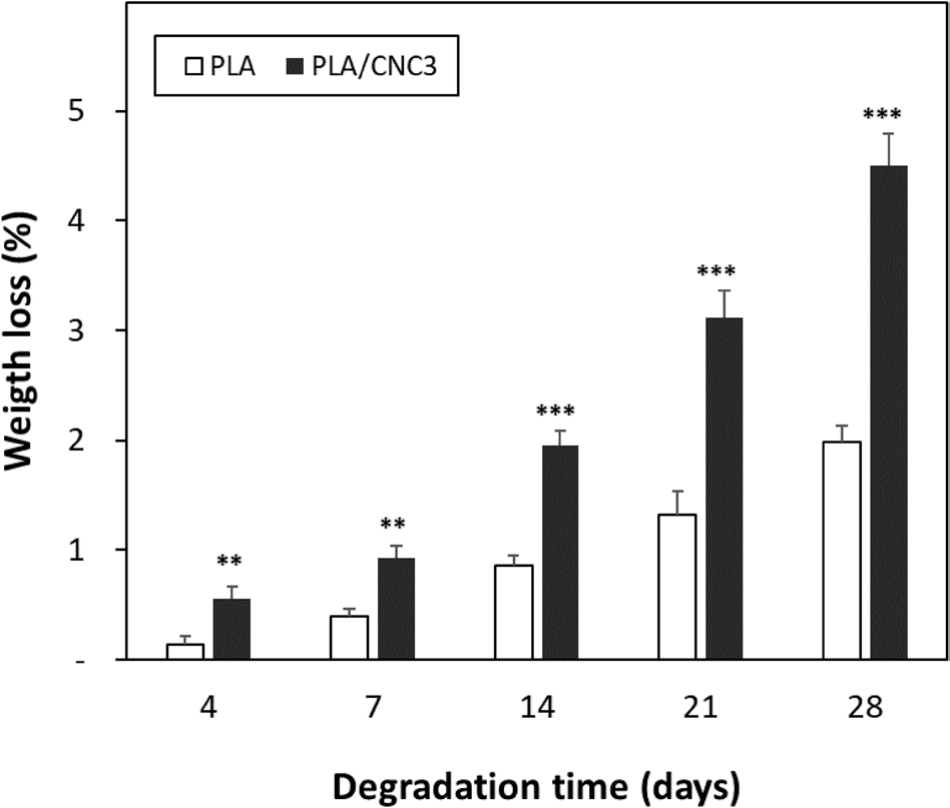
Weight loss of PLA and PLA/CNC3 scaffolds as a function of degradation time (scaffolds immersed in alcalase buffer over a period of 28 days). (**p ˂ 0.01 and ***p ˂ 0.001 using an unpaired-t-test analysis).

The enzymatic degradation of biomedical scaffolds is routinely examined by determining the weight loss. In our study, PLA and PLA/CNC3 scaffolds were subjected to an in vitro degradation study by immersing them in alcalase buffer over a period of 28 days. According to the results shown in Fig. 5, it appears that the PLA scaffold has a slower degradation compared to PLA/CNC3 composite. Indeed, PLA and PLA/CNC3 have degradation rates evaluated respectively at 1.7 ± 0.3% and 3.9 ± 0.1% after 28 days of immersion in the alcalase buffer. They are therefore considered stable. The incorporation of CNC into the PLA matrix relatively improves the degradation of PLA. Compared to studies by *Belaid* et al.^29^ on PLA and graphene oxide (PLA/GO) composite scaffolds, CNC loading leads to a moderate improvement in the degradation rate of PLA scaffolds in alcalase buffer. Thus, CNC appear as a suitable reinforcement of PLA. Therefore, PLA/CNC biocomposite can be designed as a potential material to develop scaffolds that can be used as temporary substitutes, especially for bone tissue regeneration. PLA/CNC composites could exhibit favorable transient properties by resorbing over time to provide space for newly grown bone tissue that will replace the scaffold in the body.

### Mineralization assays

**Figure 6:**
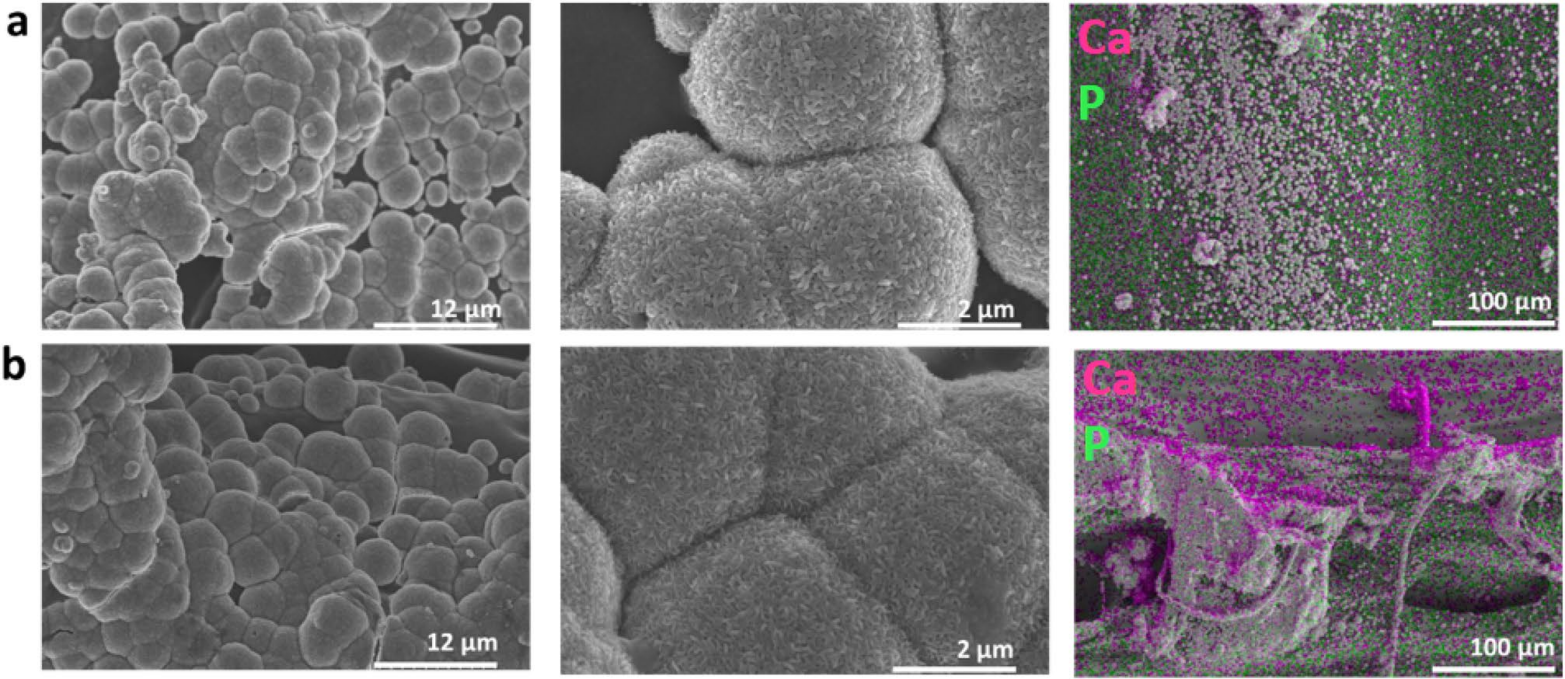
Evaluation of the apatite growth on PLA and PLA/CNC3 scaffolds mineralized in SBF at 14 days by scanning electron microscopy at high and low magnification (SEM) to validate the formation of the inorganic layer and by elemental mapping to show the presence and homogeneous distribution of the phosphate and calcium elements that constitute the apatite. (**a**) SEM images and elemental mapping of PLA, (**b**) SEM images and elemental mapping of PLA/CNC3.

The biomimetic mineralization process of printed scaffolds in SBF was chosen to evaluate the formation of apatite on the surface of PLA and PLA/CNC3 in order to conclude on their bone regeneration and binding capacity. Figure 6 shows SEM images of the surface of PLA and PLA/CNC3 scaffolds after 14 days of incubation in the SBF solution. The process of mineral growth on the surface of the scaffolds was successfully achieved by forming a bioactive coating. For all the samples, the formation of an inhomogeneous mineral layer is clearly observed, reflected by the irregular distribution of aggregates of nanocrystals on the surface, the size and density of which are dependent on the incubation time and the composition of the scaffold. Several authors have observed similar results^68–73^. The nucleated mineral particles on the surface of the scaffolds have a rod-like aspect. The mineralization of apatite crystals depends on the pH of the medium, the adsorption and release of ions at the interface and the surface wettability^71^. Overall, mineralization increases with the incubation time and is larger and faster for PLA/CNC3 scaffolds. Indeed, the deposition of apatite on the surface of PLA is relatively slow due to its slow hydrolysis in SBF solution^69^. Hydrolysis of PLA in SBF solution can take several weeks to form new carboxyl (–COOH) and hydroxyl (–OH) groups on its surface. Then the carboxyl groups undergo partial dissociation to give carboxylate ions (COO^−^) on the surface causing its negative charge. The strong electrostatic interaction between COO^−^ and Ca^2+^ ions as well as the strong hydrogen bonds between carboxyl groups and PO_4_^3−^ ions will then drive the process of accumulation of calcium Ca^2+^ and phosphate ions PO_4_^3−^^69,74^.

On the other hand, we might speculate that the addition of CNC to the PLA matrix could modify the mineral layer formation process due to hydrophilicity resulting from the formation of additional hydroxyl groups on the PLA surface. These functional groups play an important role in the ion exchange between the surface and the surrounding liquid and in the deposition of apatite on the scaffold. Indeed, these hydroxide groups bring additional negative charges to the surface of the scaffold to interact with the Ca^2+^ and PO_4_^3−^ ions of the SBF thus promoting the adsorption and the nucleation of apatite^68,71,75,76^. In view of these results, PLA/CNC composites are good candidates to support apatite formation and therefore contribute to bone regeneration and bonding by providing convenient microenvironment for cell growth and repair function.

### Cytocompatibility tests

**Figure 7:**
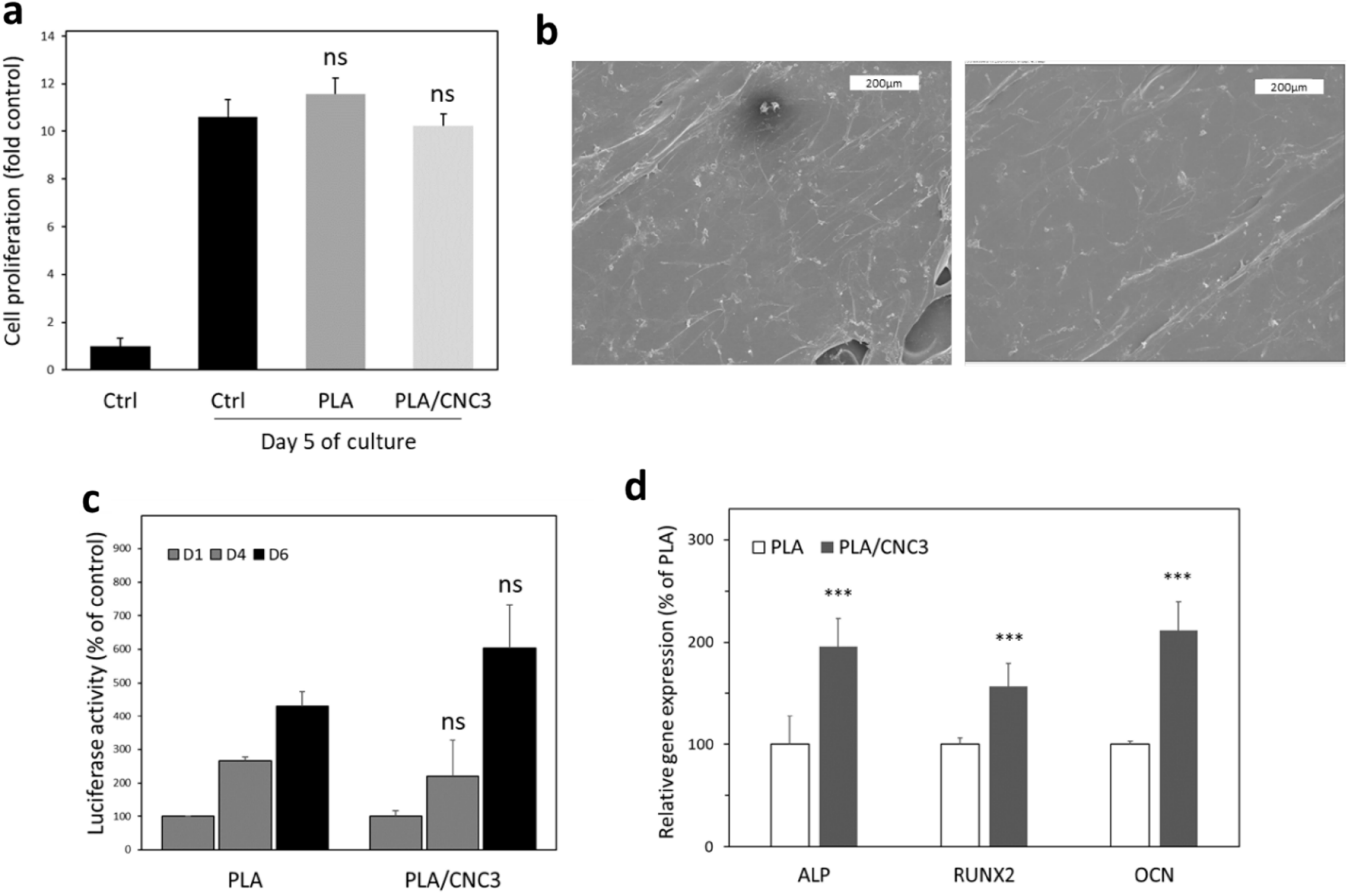
Cytocompatibility of PLA and PLA/CNC filaments and scaffolds. (**a**) Effect of PLA or PLA/CNC3 filaments on hFOB human osteoblasts viability at day 5 of culture. (**b**) Scanning electron microscopy showing hFOB cells grown on the PLA (left panel) and PLA/CNC3 (right panel) scaffolds (scale bar = 200 µm). (**c**) Growth of luminescent hFOB cells on the PLA and PLA/CNC3 scaffolds at days 1, 2 and 4. (**d**) Expression of osteogenic differentiation marker genes on hFOB cells grown for 2 days in presence of PLA and PLA/CNC3 eluates. Statistical analysis has been performed using the Mann–Whitney test between PLA and PLA/CNC3 (ns, not significant; ***, *p* < 0.001).

The biocompatibility of the PLA/CNC scaffold was investigated using hFOB1.19 human osteoblasts^77^. Compared to untreated control cells (Ctrl), incubation with PLA and PLA/CNC3 filaments showed no inhibitory effect on the cell viability at day 5 (Fig. 7a) confirming that PLA and CNC are cytocompatible.

To determine that bone cells were able to adhere and proliferate on the corresponding scaffolds, we performed scanning electron microscopy after seeding the hFOB cell onto the scaffolds. As shown in Fig. 7b, hFOB cells fully spread onto the PLA and PLA/CNC3 scaffolds and exhibited normal morphology. Additionally, we also seeded bioluminescent hFOB cells (osteoblastic cell line) onto the scaffolds and monitored their growth over time. After 6 days, the osteoblasts proliferate significantly on both scaffolds and no significant differences were observed on PLA/CNC3 scaffold as compared to PLA (Fig. 7c). Finally, we assess the effect of PLA and PLA/CNC3 on hFOB differentiation by measuring by RT-QPCR the expression of genes (ALP, RUNX2 and OCN) used as markers of bone differentiation. As shown in Fig. 7d, the level of expression of the 3 genes was higher in the presence of PLA/CNC3 than in the presence of PLA alone. Altogether, these data suggest that PLA/CNC3 scaffolds fully support bone cell growth and are biocompatible.

Overall, our approach in designing 3D biomimetic scaffolds for bone tissue engineering is in agreement with previous investigations demonstrating that reinforcing PLA with nanomaterials improves the biological and mechanical properties of the material^21,29^. Nevertheless, the existing nanofillers are synthetic and could trigger undesired effects if they accumulate in the tissues. Here, a natural biopolymer with relevant biological responses, i.e., CNC, incorporated into the PLA matrix at an optimal amount, significantly improves the mechanical strength of the scaffold without altering the tunable porosity and structure. This is a key parameter to achieve optimal functionality of state-of-the-art biomimetic tissue scaffolds.

## Conclusion

PLA scaffolds reinforced with cellulose nanocrystals have been successfully designed and manufactured using FDM technology. Their structure, surface, morphological and mechanical properties were evaluated as well as their biological performance. The 3D cellular scaffolds are highly porous with evident presence of an interconnected repetitive porous architecture favorable to the enhancement of bone tissue growth by allowing optimal vascularization, hydroxyapatite nucleation and mineral maturation. An incorporation of 3% (w/w) of CNC in the composite increases the mechanical properties of PLA and the surface roughness, while contributing to the improvement of the wettability by a surface transition of PLA from hydrophobic to hydrophilic. The mineralization process of the printed scaffolds using simulated body fluid and the nucleation of hydroxyapatite were confirmed. Finally, PLA/CNC composites appeared fully compatible with human osteoblasts and allowed their adhesion and proliferation. Our results shed light on the relevance of CNC in PLA-based composites for tissue engineering and open simple and promising avenues to design multifunctional 3D printed scaffolds that are active for future tissue regeneration applications.

## Methods

### Materials

Polylactic acid (PLA) pellets were purchased from NatureWorks LLC. Dichloromethane (CH_2_Cl_2_, < 99.7%, CAS 75-09-2, Sigma-Aldrich), sodium hydroxide (NaOH, pellets, 98.9%, Sigma-Aldrich), sulfuric acid (H_2_SO_4_, 95.0–98.0%, CAS 7664-93-9, Sigma-Aldrich), hydrogen peroxide (H_2_O_2_, 30% (w/w), CAS 7722-84-1, Sigma-Aldrich), ethanol (96% vol, CAS 64-17-5, Sigma-Aldrich), sodium chloride (NaCl, ≥ 99%, CAS 7747-14-5, Sigma-Aldrich), sodium hydrogen carbonate (NaHCO_3_, ≥ 99.7%, CAS 144-55-8, Sigma-Aldrich), potassium chloride (KCl, ≥ 99,7%, CAS 7447-40-7, Sigma-Aldrich ), di-potassium hydrogen phosphate trihydrate (K_2_HPO_4_.3H_2_0, ≥ 99%, CAS 16788-57-1, Sigma-Aldrich) magnesium chloride hexahydrate (MgCl_2_.6H_2_O, ≥ 99%, CAS 7791-18-6, Sigma-Aldrich), calcium chloride (CaCl_2_, ≥ 97%, CAS 10043-52-4, Sigma-Aldrich), sodium sulfate (Na_2_SO_4_, ≥ 99.0%, CAS 7757-82-6, Sigma-Aldrich), Tris-hydroxymethyl aminomethane ((HOCH_2)3_CNH_2_) (tris), ≥ 99.8%, CAS 1185-53-1, Sigma-Aldrich), hydrochloric acid (HCl, 36% CAS 7647-01-0, Sigma-Aldrich), Bacillus licheniformis (alcalase, CAS 126741 Sigma-Aldrich), L-cysteine (CAS,52-90-4 ), Azide sodium (CAS 26628-22-8, Sigma-Aldrich), TRIS buffer pH 9.0 (CAS 77-86-1, Alfa Aesar), Dimethyl sulfoxide (DMSO, CAS 23486.297; BDH Prolab), Dulbecco’s Modified Eagle Medium α (DMEM/F12, CAS 10565018; Gibco)), 3-(4,5-Dimethylthiazol-2-yl)-2,5-Diphenyltetrazolium Bromide (MTT, 98% , CAS 298-93-1, Sigma-Aldrich), Fetal bovine serum (FBS, CVFSVF00-01; Eurobio), Trypsin-EDTA (CAS 25300-054; Gibco), hexamethyldisilazane (CAS 999-97-3, Sigma-Aldrich), were obtained and used without further purification. hFOB osteoblastic cell line obtained from ATCC was used for cytocompatibility analysis.

### Cellulose nanocrystals synthesis

Cellulose nanocrystals (CNC) were synthesized from *Ficus thonningii* (FT) by a three-step method based on (1) alkali treatment, (2) bleaching and (3) acid hydrolysis as proposed by Dingyuan Zheng^78^ with modifications. Briefly, FT raw fibers, previously dried and stored at 70 °C, were cut, crushed and then dispersed in an alkaline solution of sodium hydroxide (NaOH, 0.5 N) with FTFs/NaOH fiber to solution ratio of 3 g/80 mL. They were stirred continuously at 100 °C for 4 h. After the alkali treatment, the fibers were filtered, washed with 18 M MilliQ water until pH neutralization. The resulting fibers were bleached with hydrogen peroxide (H_2_O_2_) for 1 h at 80 °C. The bleaching sequence was repeated several times to obtain brilliantly white fibers. Finally, nanocellulose synthesis was performed by the acid hydrolysis process followed by ultrasound sonication. In details, 10 g of bleached fibers were dissolved in 200 g of an aqueous solution of sulfuric acid (64%) with magnetic stirring for 1 h 30 min at 45 °C. Then, the suspension obtained was filtered, washed with 18 M MilliQ water several times and was then subjected to dialysis for 7 days (in bags of dialysis from 12,000 to 14,000 Da). After dialysis, the resulting solution was sonicated in an ice bath using an ultrasonic homogenizer (BANDELIN electronic, GM 3100) fitted with a 7 cm probe tip. The operating range of the ultrasonic homogenizer was set at 50%. The resulting suspension was centrifuged at 10,000 rpm for 30 min and the paste was frozen, lyophilized and then ground using a Moulinex mixer (LM935) at 8 000 rpm for 3 min to obtain a white powder of cellulose nanocrystals (CNC). Figure S5 summarizes the steps of the cellulose nanocrystals synthesis from FT stem (See Supplementary Information).

### Filaments extrusion and 3D printing of PLA/CNC scaffolds

**Figure 8:**
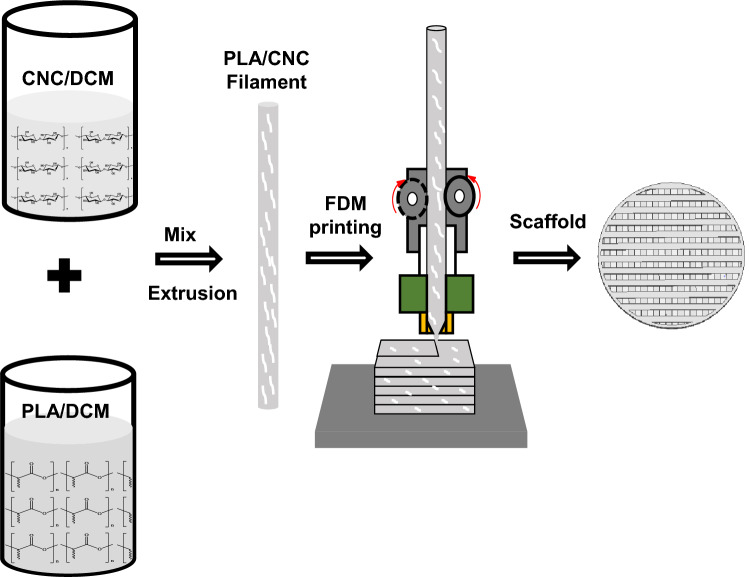
Fabrication steps of PLA/CNCx scaffold (extrusion of the composite filament and 3D printing).

In order to produce homogeneous PLA/CNC composite filaments by single-screw extrusion, we initially proposed to formulate composites by incorporating nanocellulose fillers into the PLA matrix by chemical dissolution. 30 g of PLA were dissolved in 200 mL of dichloromethane (DCM) at room temperature with continuous magnetic stirring followed by sonication in an ice bath for 5 min. Then, the suspension of CNC/DCM was added to that of PLA to give blend slurries of PLA/CNC/DCM with 0, 1, 3 and 5% (w/w) of CNC relative to PLA (Table 3). The PLA/CNC/DCM suspensions obtained were sonicated in an ice bath using an ultrasonic homogenizer for 15 min and kept under continuous magnetic stirring at room temperature for 24 h to ensure good homogenization. They were then left to dry overnight at room temperature, then cut into small pieces and dried under vacuum for 24 h to ensure complete drying. The resulting dried composite polymers were extruded using a Noztek model MHB26234 single screw extruder at a temperature range of 160–165 °C. They were then cooled by conversion at the nozzle outlet using the extruder fan to give PLA/CNC hybrid filaments denoted PLA/CNCx, where x corresponds to the content of CNC. Finally, filaments of approximately 1.75 ± 0.05 mm in diameter were selected for 3D printing. The pure PLA filaments were produced by direct extrusion from PLA scoops under the same conditions as the composite filaments. Figure 8 summarizes the preparation of PLA/CNC composite filaments. The extrusion sequence was repeated 2 to 3 times to obtain PLA/CNCx filaments of homogeneous diameters. The scaffold was modeled by Computer Aided Design (CAD) using Design Spark Mechanical software, translated to STL file and sliced by Prusa3Dslicer software to generate the G-code. Finally, the cylindrical designs (porous) (diameter (10 mm) × height (2 mm)) and the rectangular pieces (dense) were designed using an FDM Prusa MK2S Research model printer equipped with a nozzle of 0,4 mm diameter at 200 °C. The filaments were deposited with a straight filling architecture in precise directions of 0° and 90° between two successive layers. The thickness of each layer was 0.15 mm with 80% filling for the cylinders and 100% filling for the rectangular pieces and the printing speed was 30 mm/s.

**Table 3:** Formulation of PLA/CNC composites.

Notation	Composition	PLA weight (g)	CNC weight (g)
PLA	100% PLA + 0% CNC	30	0.0
PLA/CNC1	99% PLA + 1% CNC	29.7	0.3
PLA/CNC3	97% PLA + 3% CNC	29.1	0.9
PLA/CNC5	95% PLA + 5% CNC	28.5	1.5

### Thermal properties

PLA and PLA/CNCx composites were examined using differential scanning calorimeter (DSC) (Q20, TA instruments), equipped with an RCS90 cooling system (TA instruments). Samples were weighed in an aluminum TA pan and sealed. An empty sealed pan was used as reference. Samples were first cooled to 25 °C and then heated to 250 °C with a heating rate of 20 °C min^−1^ under nitrogen atmosphere. The thermal properties of PLA and PLA/CNCx composites such as the glass transition temperature (T_g_), the cold crystallization temperature (Tcc), the melting temperature (Tm) and the enthalpy of fusion (Δ*H*m) were evaluated by the heating scans. The degree of crystallinity (${\chi }_{c}$) was determined for both the cold crystallization peak and the melting peak using the following equations^56^:

1 $$\chi_{c} = \frac{{\Delta H_{cc} }}{{\Delta H_{m0} }}*\frac{100}{W}$$

where Δ*H*_cc_ is the enthalpy of cold crystallization, *W* is the weight fraction of PLA in the sample and Δ*H*_m0_ is the enthalpy of melting for 100% crystalline PLA material, which was taken as 93 J g^−1^. The thermogravimetric analysis (TGA) was performed using a TGA Q500 device (TA instruments). 10 mg of each sample were heated under nitrogen from room temperature to 800 °C at a heating rate of 10 °C min^−1^.

### Structural and morphological characterization

The Fourier transform infrared (FTIR) spectroscopy was performed in an attenuated total reflectance mode (ATR-FTIR) on a NICOLET NEXUS model spectrometer. All spectra were recorded in a spectral range of 4000–650 cm^−1^ with an accumulation of 32 scans at a resolution of 4 cm^−1^.

The morphology of CNC, extruded PLA and composite filaments as well as 3D printed scaffolds were observed using HITACHI S4800 scanning electron microscopy. The samples were sputter-coated for 30 s with platinum using a Polaron SC7620 Mini Sputter Coater for SEM analysis. Image J software was employed to calculate the mean filament diameter by taking average at 20 points and the pore size by taking average of 8 pores, which is denoted as mean ± standard deviation. Topography of the scaffolds’ surfaces was analyzed using 3D optical microscopy (Keyence) and a confocal chromatic roughness tester (STIL SA) equipped with a CHR1000 sensor.

The porosity of the scaffold was obtained by a liquid displacement method as reported in the literature^64^. Briefly, the scaffolds were immersed in tubes containing a specific amount of ethanol (*W*_1_) for 30 min. Then, the total weight of immersed scaffolds and ethanol was noted as *W*_2_. After removing the scaffolds, the residual ethanol in the tubes was noted as *W*_3_. The porosity of the scaffolds was measured according to the following equation:

2 $$Porosity \left( \% \right) = 100*\frac{{W_{1} - W_{3} }}{{W_{2} - W_{3} }}$$

### Mechanical properties

The mechanical properties of 3D printed PLA/CNC scaffolds were characterized using a tensile system (Zwick Roell), coupled with a 5KN load cell. The specimens were printed in the form of a dog bone (40 mm long, 4 mm wide and 1.5 mm thick). The specimens were then clamped between dedicated jaws and pulled at a speed of 0.05 mm s^−1^ until they broke. The Zwick Roell software is able to calculate Young’s modulus, maximum force at break and elongation at break. At least 5 specimens are printed under the same conditions in order to perform a statistical measurement.

### Contact angle and swelling

The contact angle of the 3D printed scaffolds with water was examined to determine their hydrophilicity at room temperature using the sessile drop method. Two scaffolds were analyzed: PLA as the reference & PLA/CNC3 as the composite with the best mechanical performance. The drops were photographed using a Drop Shape analyzer—DSA25 equipped with a monochrome camera B-CAM-21-BW (CCCIR) and an R60 Led lamp (Conrad). Briefly, 6 μL of 18 M MilliQ water was dropped onto the surface of each scaffold and the contact angle at equilibrium (considered at 60 s) was recorded. One Touch Grabber and Image J software were used to calculate the contact angles. The swelling behavior of the scaffolds was determined by gravimetric method. Scaffolds of previously known weight (*W*_d_) are immersed in 10 mL of 18 M MilliQ water, then incubated with continuous stirring at 37 °C for an equilibrium time assumed to be 7 days. Then the samples were removed from the water, cleaned with filter paper to remove excess residual water adsorbed on the surface and weighed (*W*_h_). Finally, the scaffold swelling index was determined as follows:

3 $$Swelling\;index \left( \% \right) = 100*\frac{{W_{h} - W_{d} }}{{W_{d} }}$$

### Enzymatic degradation

The enzymatic degradation of PLA and PLA/CNC3 was carried out using alcalase enzyme according to a protocol previously reported in the literature with modifications^29,79,80^. Indeed, strips of PLA and PLA/CNC3 tissues (dimensions 5 [W] × 15 [l] × 0.13 [h]) of approximately 0.25 g were printed and immersed in 25 mL of TRIS buffer (pH 9.5, 60 °C) with an optimum concentration of 50% (w/w) (relative to the weight of the tissue) of alcalase required for enzymatic hydrolysis, 3 mM L-cysteine and 0.05% (w/w) of azide sodium (relative to the weight of the TRIS buffer). Degradation was assessed by determining weight loss at 4, 7, 14, 21 and 28 days. The samples were first dried at 105 °C for 90 min, cooled in a desiccator then weighed in a closed weighing bottle. The percentage of weight loss was calculated as follows:

4 $$Weight\;loss \left( \% \right) = 100*\frac{{W_{1} - W_{2} }}{{W_{1} }}$$

where *W*_1_ and *W*_2_ are the dry weight of the samples before and after biodegradation, respectively.

### Cell viability

The osteoblastic cell line hFOB 1.19 (ATCC® CRL11372™, ATCC, USA)^77^ was cultured using DMEM/F12 (Dulbecco’s Modified Eagle Medium α) (Gibco 10565018) conditioned media supplemented with 10% (V/V) foetal bovine serum (FBS) (Eurobio CVFSVF00-01). Cells were cultured at 37 °C in 5% CO_2_ in a 10 cm diameter petri dish and trypsinized using 0.05% Trypsin–EDTA (Gibco 25300-054). After sterilization with 70% (w/v) ethanol for 30 min and UV irradiation for 1 h, the filaments and printed scaffolds were dried at room temperature and then placed in contact with hFOB cells for 5 days.

Cell viability was analyzed using MTT assay carried out by incubating 100 µL of 0.5 mg/mL of MTT solution on the cells for 3 h. Purple coloured formazan crystals were dissolved using 100 µL of DMSO (BDH Prolab 23486.297) and the absorbance was recorded at 560 nm using Multiskan plat reader (thermos, USA).

### Adhesion assays

For adhesion assays, hFOB 1.19 cells were cultured on the scaffolds for 4 days (seeding of 2 × 10^4^ cells per well of 24 well plate containing the scaffold). Cells cultured on scaffolds were fixed with 2.5% glutaraldehyde solution for 2 h at room temperature. Scaffolds with cells were then incubated twice for 15 min at room temperature, using increasing ethanol concentrations (30%, 50%, 70%, 95% (v/v)), followed by absolute ethanol then hexamethyldisilazane overnight. For scaffold growth assays, luciferase expressing hFOB 1.19 cells were cultured on scaffolds for 6 days (seeding of 2 × 10^4^ cells per well of 24 well plate containing the scaffold). Luciferase activity was quantified using a Luminometer after cell lysis. In order to investigate only the influence of cellulose on PLA biological properties, a 2D model was used.

### Gene expression analysis

hFOB 1.19 cells were seeded at 500 000 cells/well in 6-well flat-bottom plates and incubated at 37 °C/5% CO_2_ for 48 h in 2 ml of medium previously incubated for 24 h with the corresponding scaffolds at 0.2 g/ml. Total RNA was extracted using the EZNA Total RNA Kit I, following the manufacturer's instructions. The cDNA was synthesized from 1 µg of RNA by reverse transcriptase (Mix qScript, Invitrogen Life Technologies). PCR reactions were performed in a thermal cycler (LightCycler® 480) for 30 cycles. The primers used are provided in Table 4.

**Table 4:** Description of the primers used for the gene expression analysis.

Gene	Forward primer	Reverse primer
ALP	5′ GCTGGCAGTGGTCAGATGTT 3′	5′ CTATCCTGGCTCCGTGCTC 3′
OCN	5′ TTGGACACAAAGGCTGCAC 3′	5′ CTCACACTCCTCGCCCTATT 3′
RunX2	5′ CCTAAATCACTGAGGCGGTC 3′	5′ CAGTAGATGGACCTCGGGAA 3′

### Mineralization assays

To assess bone bioactivity, the ability to form apatite on PLA and PLA/CNC3 scaffolds was studied by mineralized culture in vitro in simulated body fluid (SBF) according to the method described in the literature^81^. Briefly, the scaffolds were immersed in 10 mL of fresh SBF and then incubated in a thermoplastic incubator shaker at 37 °C. The SBF solution was changed every other day throughout the 4-weeks study period. After incubation, weekly samples were removed from the fluid and dried in a desiccator. Finally, scanning electron microscopy (SEM) and Energy Dispersive X-ray spectroscopy (Detector: Oxford Instruments X-Max AZTEC, UK; Microscopy: Zeiss EVO HD15, Germany) were carried out to investigate the degree of mineralization of the scaffolds.

### Statistical analysis

For most of the quantitative characterization method, the ANOVA test was used to evaluate if the data from every group of samples showed a significant difference (*p* < 0.05 for significant). For the degradation assay, an unpaired-t-test was performed to compare data from PLA to PLA/CNC3. For the biological tests, the Mann–Whitney test was performed for the statistical analysis.

## Supplementary Information

Supplementary Information.

## Data Availability

All data generated or analyzed during this study are included in this published article (and its Supplementary Information files).
