# Supplementary material for: 3D printing of cellulose nanocrystals based composites to build robust biomimetic scaffolds for bone tissue engineering
Source: Sci Rep. 2022 Dec 8;12:21244. doi: 10.1038/s41598-022-25652-x (PMC9732347; doi:10.1038/s41598-022-25652-x)
Supplement: Supplementary file 1 — Supplementary Information. [file 41598_2022_25652_MOESM1_ESM.pdf]

Supplementary Information for:

3D printing of cellulose nanocrystals based composites to build robust biomimetic scaffolds for bone tissue engineering

Kanga Marius N'Gatta[1,2], Habib Belaid[1,3], Joelle El Hayek[1], Edja Florentin Assanvo[2], Marilyn Kajdan[3], Nathalie Masquelez[1], David Boa[2], Vincent Cavaillès[3], Mikhael Bechelany[1], Chrystelle Salameh*[1]

[1] Institut Européen des Membranes, IEM, UMR 5635, Univ Montpellier, ENSCM, CNRS, Montpellier, France

[2] Laboratoire de Thermodynamique et de Physico-Chimie du Milieu, UFR SFA, Université Nangui Abrogoua, 02 BP 801 Abidjan 02, Côte d'Ivoire

[3] IRCM, Institut de Recherche en Cancérologie de Montpellier, INSERM U1194, Université Montpellier, Montpellier F-34298, France

*Corresponding author : chrystelle.salameh@enscm.fr (Chrystelle Salameh).

FTIR spectroscopy of PLA and PLA/CNCx filaments

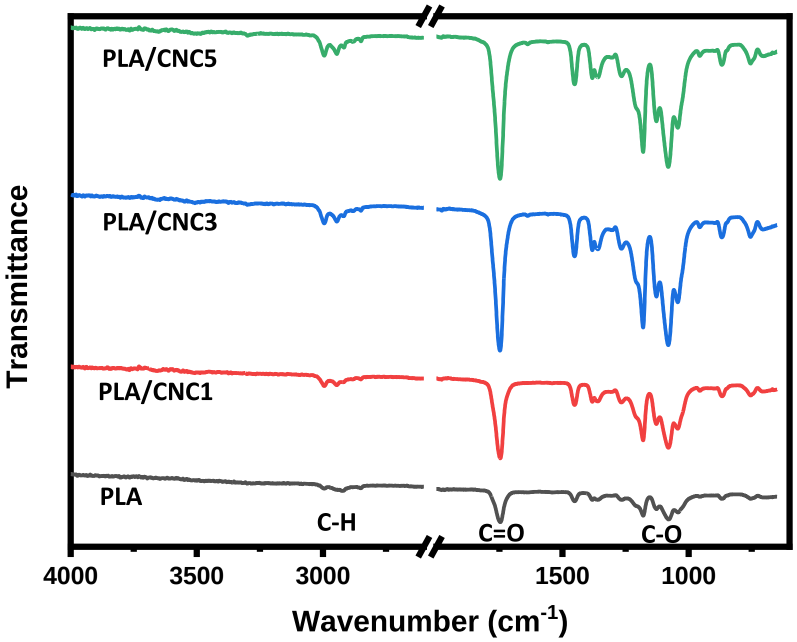

Figure S1. FTIR spectra of PLA and PLA/CNCx composite filaments.

Thermal analysis of PLA/CNCx composites and their constituents

The thermal characteristics of CNC were examined based on the results of TGA and DTG shown in **Figure S2**. The CNC degradation kinetics observed on the TG curve shows three stages of weight loss in the temperature range of 25 to 800°C. Initially, from approximately 25 to 200°C, a slight weight loss of 3.78% observed is attributed to to the drying step[1]. The second weight loss is observed in the range of 200 to 400 °C, which could be related to the depolymerization or the breaking of the glycosidic bonds of the cellulose[2,3,4]. The actual degradation starts around 250°C. The maximum temperature (Tmax= 370°C) of the peak clearly observed on the derived profile can be assigned to the maximum degradation temperature of the synthesized nanocellulose.

From the results of the TG & DTG analyses, it is evident that CNC is thermally stable and therefore can be used as a reinforcement in polymer matrices with operating temperatures below 370°C.

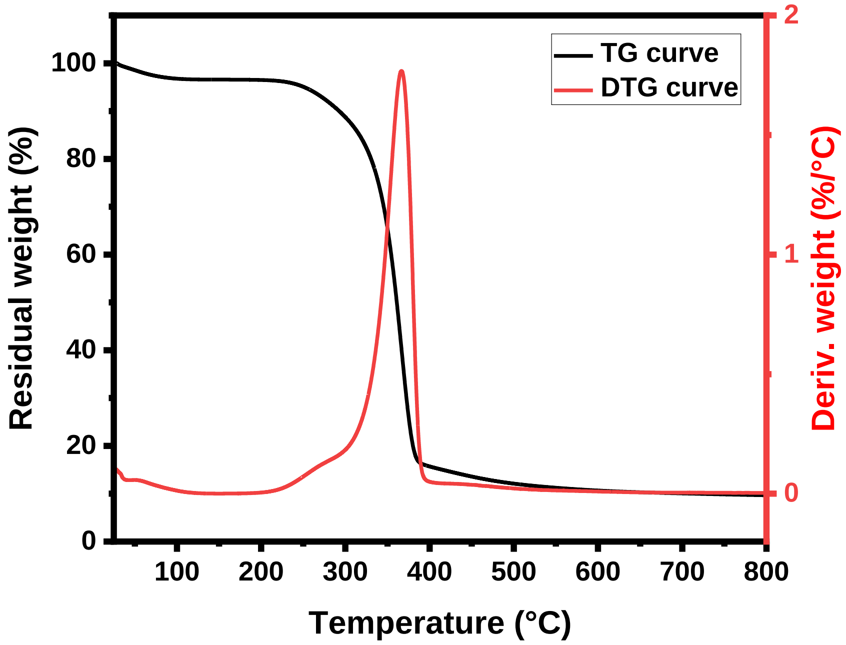

Figure S2. Thermal analysis of CNC.

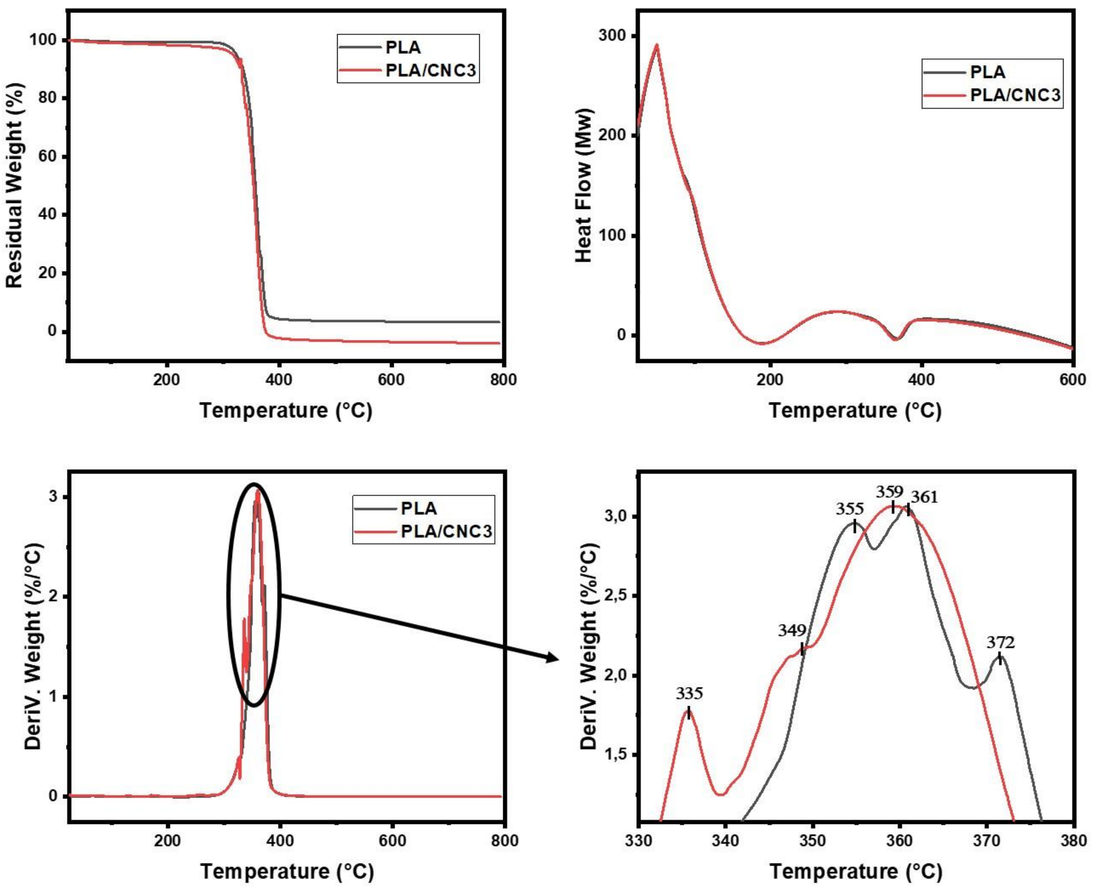

Figure S3. Thermal analysis of 3D printed PLA and PLA/CNC3.

Contact angle measurements of PLA and PLA/CNC3

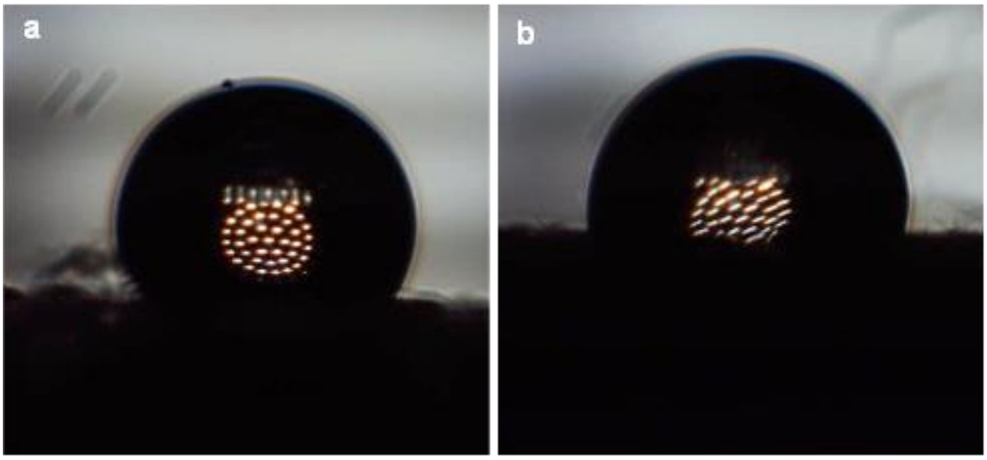

Figure S4. Contact angle images of water on (a) PLA and (b) PLA/CNC3.

Experimental section

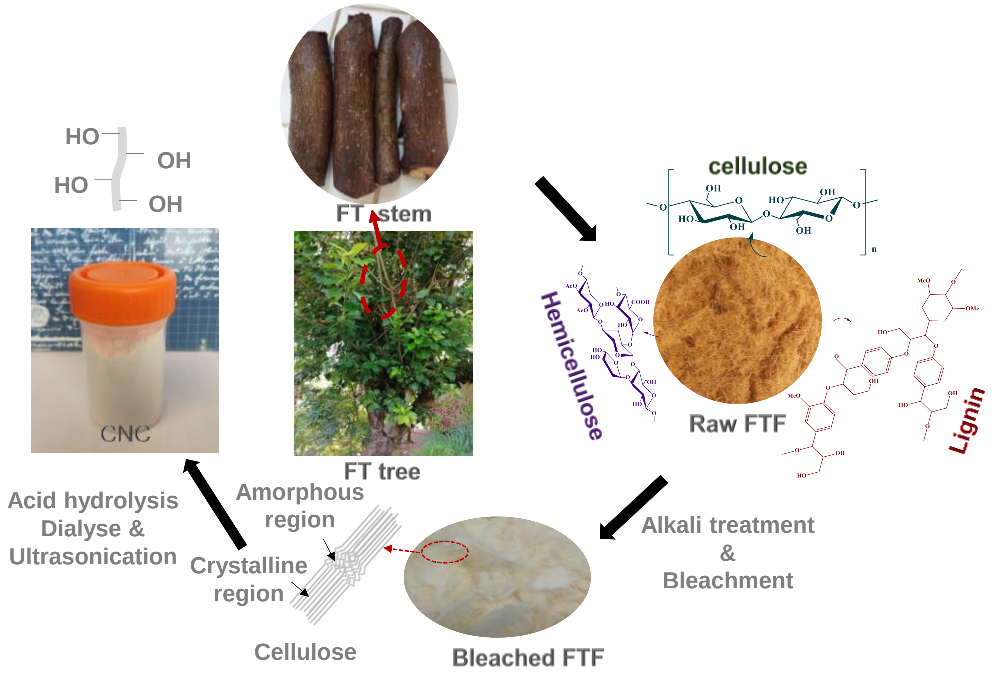

Figure S5. Synthesis of cellulose nanocrystals from Ficus thonningii (FT) stem.

References

1. Onkarappa, H. S. *et al.* Hevea brasiliensis mediated synthesis of nanocellulose : Effect of preparation methods on morphology and properties. *Int. J. Biol. Macromol.* **160**, 1021–1028 (2020).
2. Kathirselvam, M., Kumaravel, A., Arthanarieswaran, V. P. & Saravanakumar, S. S. Isolation and characterization of cellulose fibers from Thespesia populnea barks: A study on physicochemical and structural properties. *Int. J. Biol. Macromol.* **129**, 396–406 (2019).
3. Kakati, N., Assanvo, E. F. & Kalita, D. Alkalinization and graft copolymerization of pineapple leaf fiber cellulose and evaluation of physic-chemical properties. *Polym. Compos.* **40**, 1395–1403 (2019).
4. Werner, K., Pommer, L. & Broström, M. Thermal decomposition of hemicelluloses. *J. Anal. Appl. Pyrolysis* **110**, 130–137 (2014).
